# Neuromelanin-sensitive magnetic resonance imaging in the diagnosis and assessment of Parkinson’s disease

**DOI:** 10.3389/fneur.2026.1908455

**Published:** 2026-10-05

**Authors:** Die Zhang, Aishanjiang Yusufujiang, Hongyan Li

**Affiliations:** 1 Department of Neurology, People’s Hospital of Xinjiang Uygur Autonomous Region, Xinjiang, China; 2 Xinjiang Clinical Research Center for Stroke and Rare Neurological Disease, Ürümqi, China

**Keywords:** imaging biomarker, multimodal imaging, neuromelanin-sensitive magnetic resonance imaging, Parkinson’s disease, substantia nigra

## Abstract

Parkinson’s disease (PD) is a progressive neurodegenerative disorder characterized primarily by the progressive loss of dopaminergic neurons in the substantia nigra pars compacta (SNpc); noradrenergic neurons in the locus coeruleus (LC) are also frequently affected. Because clinical motor symptoms usually emerge only after substantial neurodegeneration has occurred, there is a compelling need for noninvasive imaging biomarkers capable of detecting early pathological changes. Neuromelanin-sensitive magnetic resonance imaging (NM-MRI) generates neuromelanin-related image contrast in the SNpc and LC and can indirectly reflect the structural integrity of these catecholaminergic nuclei. Current evidence suggests that NM-MRI may be useful for distinguishing patients with PD from healthy controls, supporting the differential diagnosis of selected atypical parkinsonian syndromes and essential tremor, assessing disease stage, monitoring longitudinal change, and evaluating high-risk individuals in the prodromal phase. This review provides an updated account of recent advances in the use of NM-MRI for diagnostic support, differential diagnosis, prodromal assessment, longitudinal monitoring, multimodal imaging, and artificial intelligence-based analysis, and discusses its clinical role relative to other imaging and fluid biomarkers. Combining NM-MRI with complementary imaging measures, such as quantitative susceptibility mapping and diffusion MRI, and with automated analytical methods may further enhance pathological characterization and quantitative assessment. However, the available evidence is derived largely from single-center or case–control studies with limited sample sizes. Substantial variability may arise from differences in scanners, magnetic field strengths, acquisition protocols, and analytical methods, while independent external validation and prospective multicenter longitudinal evidence remain limited. The biological basis of the NM-MRI signal has not been fully established, and artificial intelligence models developed for NM-MRI remain vulnerable to overfitting, limited interpretability, and limited cross-center generalizability. Accordingly, NM-MRI cannot currently serve as a stand-alone diagnostic tool for PD or replace diagnosis based on comprehensive clinical assessment. It is better regarded as a promising complementary imaging biomarker and research tool. Before NM-MRI can be widely used for routine clinical diagnosis, disease monitoring, or as a clinical trial outcome measure, acquisition and analytical methods must be further standardized, and its performance must be evaluated in large, prospective, multicenter longitudinal studies.

## 1 Introduction

Parkinson’s disease (PD) is a progressive neurodegenerative disorder characterized primarily by the gradual loss of dopaminergic neurons in the substantia nigra pars compacta (SNpc); noradrenergic neurons in the locus coeruleus (LC) are also frequently affected (1). The diagnosis of PD continues to rely heavily on clinical motor manifestations, by which time substantial degeneration of nigral dopaminergic neurons has usually already occurred (2). This diagnostic delay underscores the need for reliable, noninvasive biomarkers to support early identification, risk stratification, and enrollment in disease-modifying clinical trials (3).

Against this background, neuromelanin-sensitive magnetic resonance imaging (NM-MRI) has attracted attention as a promising imaging biomarker. It generates neuromelanin-related image contrast in the SNpc and LC and can indirectly reflect the structural integrity of these catecholaminergic nuclei (2, 4). NM-MRI does not directly quantify neuromelanin, and its signal mechanism remains debated. Potential contributors include T1-shortening effects associated with neuromelanin and neuromelanin-iron complexes, differences in tissue proton density and water content, and magnetization transfer effects. Recent longitudinal evidence spanning animal models, histology, and human cohorts has shown an initial signal increase with intracellular neuromelanin accumulation followed by a decline with neurodegeneration, supporting an important contribution from neuromelanin-related T1 shortening. However, the relative contribution of each mechanism across sequences, ages, and disease stages remains unclear (2, 4, 69). Current findings suggest that NM-MRI may have complementary value for diagnostic support, differential diagnosis, and disease-progression monitoring in PD, although its clinical performance requires further validation (1, 5).

In clinical and research settings, NM-MRI may provide complementary information across several dimensions. First, as an MRI method that does not involve ionizing radiation, NM-MRI generates neuromelanin-related image contrast in the SNpc and indirectly reflects local structural integrity, making it a potential complement to dopamine transporter (DAT) imaging (6, 7). Second, when combined with iron-sensitive imaging and diffusion MRI, NM-MRI may provide complementary biomarker information (1, 3). Its potential roles in prodromal assessment of high-risk populations and as an outcome measure in disease-modifying clinical trials are also being investigated, but require further validation (8–12).

Although the basic imaging principles of NM-MRI and its preliminary applications in PD have been discussed extensively, several aspects of the current evidence require further integration. First, the maturity of the evidence varies across clinical scenarios. Studies distinguishing established PD from healthy controls are relatively numerous, whereas evidence for prodromal detection, individualized prediction of progression, and use as a clinical trial outcome measure remains limited. Second, the clinical role of NM-MRI relative to DAT imaging, quantitative susceptibility mapping (QSM), diffusion MRI, functional imaging, fluid biomarkers, and *α*-synuclein seed amplification assays (SAAs) has not been fully defined. Finally, although multimodal and artificial intelligence models have reported high diagnostic performance, they differ substantially in sample size, validation strategy, cross-center generalizability, and feasibility of clinical deployment. Accordingly, in addition to briefly outlining the technical basis of NM-MRI, this review updates recent advances and systematically summarizes the evidence for diagnostic support, differential diagnosis, prodromal assessment, longitudinal monitoring, and clinical trial applications. It also details the sample composition, input features, validation strategies, and performance metrics of multimodal and artificial intelligence studies. The principal contribution of this review is to move beyond the diagnostic performance of individual measures and address the clinical role of NM-MRI, the maturity of its supporting evidence, and barriers to translation, together with its complementary relationships with other imaging and fluid biomarkers. To summarize these themes visually and clarify the current clinical role of NM-MRI, Figure 1 presents a six-panel framework encompassing the biological basis of NM-MRI, its principal anatomical targets, conventional and emerging imaging approaches, clinical and research applications, multimodal biomarker integration, as well as its current limitations, future priorities, and clinical positioning.

**Figure 1:**
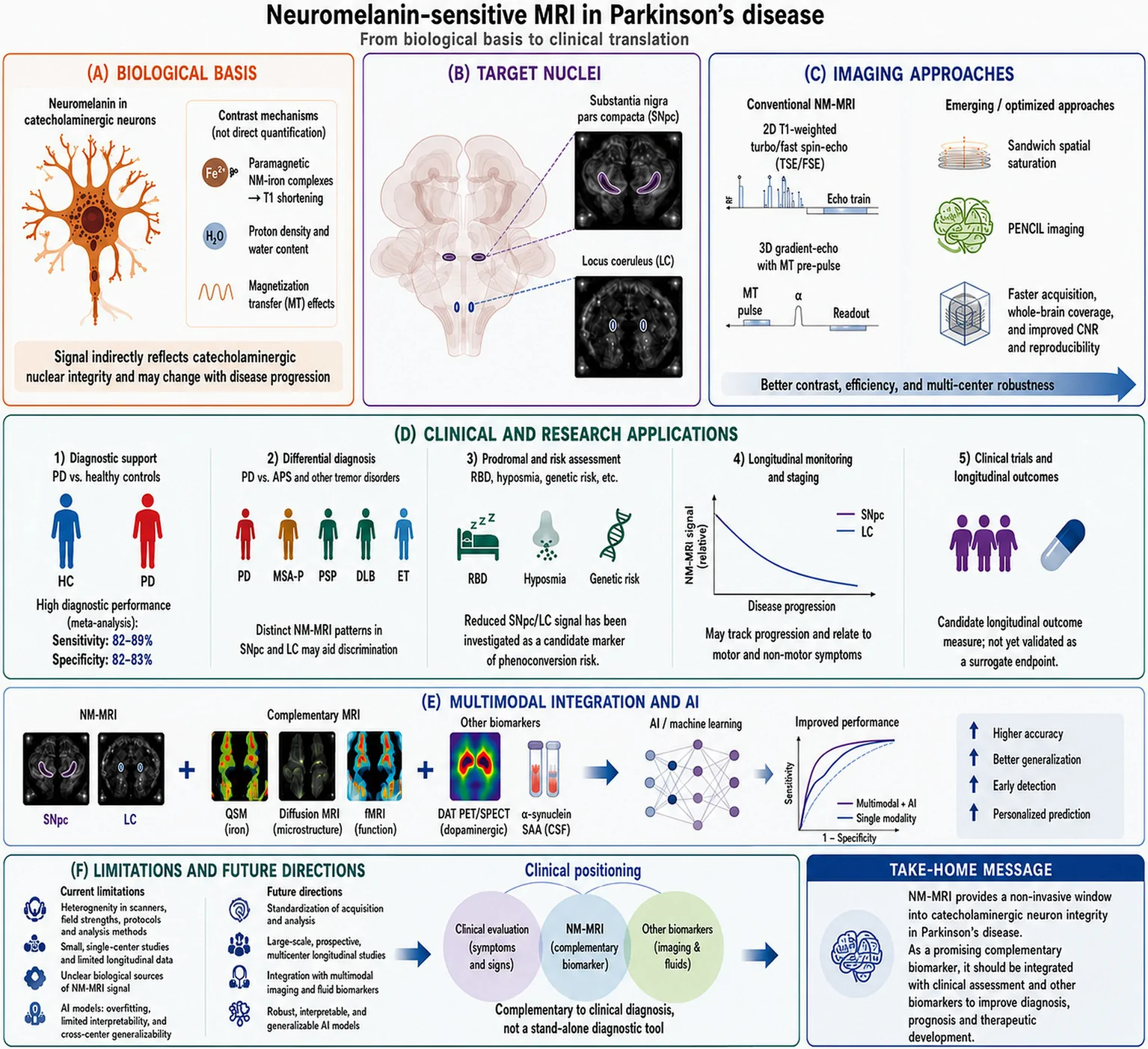
Biological basis, anatomical targets, imaging approaches, and potential clinical applications of neuromelanin-sensitive MRI in Parkinson’s disease. **(A)** Neuromelanin in catecholaminergic neurons contributes to neuromelanin-sensitive MRI (NM-MRI) contrast through T1 shortening associated with paramagnetic neuromelanin–iron complexes, differences in proton density and water content, and magnetization-transfer effects. NM-MRI provides an indirect marker of catecholaminergic nuclear integrity rather than a direct quantitative measurement of neuromelanin. **(B)** The principal target nuclei are the substantia nigra pars compacta (SNpc), located in the ventral midbrain dorsal to the cerebral peduncles, and the locus coeruleus (LC), located in the rostral dorsal pons adjacent to the floor of the fourth ventricle. The images are schematic representations and are not derived from individual patients. **(C)** Imaging approaches include two-dimensional T1-weighted turbo/fast spin-echo imaging, three-dimensional gradient-echo imaging with magnetization-transfer preparation, sandwich spatial saturation, and PENCIL imaging. These methods aim to improve contrast, acquisition efficiency, brain coverage, contrast-to-noise ratio, reproducibility, and multicenter robustness. **(D)** Investigated applications include diagnostic support, differential diagnosis, prodromal and high-risk assessment, longitudinal monitoring and staging, and clinical trials. Meta-analyses have reported relatively high diagnostic performance for differentiating Parkinson’s disease (PD) from healthy controls, although acquisition and analysis methods and diagnostic thresholds remain heterogeneous. Distinct SNpc and LC patterns may assist differential diagnosis. Reduced SNpc and LC signals have been investigated as candidate markers of phenoconversion risk. NM-MRI is also being studied as a candidate longitudinal outcome measure but has not been validated as a surrogate endpoint. **(E)** Multimodal integration may combine NM-MRI with quantitative susceptibility mapping, diffusion MRI, functional MRI, dopaminergic PET/SPECT, cerebrospinal fluid *α*-synuclein seed amplification assays, and artificial intelligence methods. Such approaches require independent external validation and prospective evaluation. **(F)** Current limitations include scanner and protocol heterogeneity, small single-center cohorts, limited longitudinal evidence, uncertain biological determinants of NM-MRI signal, and risks of model overfitting and limited generalizability. Future priorities include acquisition and analysis standardization, large prospective multicenter studies, integration with complementary biomarkers, and robust, interpretable artificial intelligence models. NM-MRI should currently be regarded as complementary to clinical evaluation and other biomarkers, not as a stand-alone diagnostic test. AI, artificial intelligence; APS, atypical Parkinsonian syndromes; CNR, contrast-to-noise ratio; CSF, cerebrospinal fluid; DAT, dopamine transporter; DLB, dementia with Lewy bodies; ET, essential tremor; FSE, fast spin echo; fMRI, functional magnetic resonance imaging; GRE, gradient echo; HC, healthy controls; LC, locus coeruleus; MSA-P, multiple system atrophy, Parkinsonian type; MT, magnetization transfer; NM-MRI, neuromelanin-sensitive magnetic resonance imaging; PD, Parkinson’s disease; PENCIL, Proton-density Enhanced Neuromelanin Contrast in Low flip-angle gradient-echo imaging; PET, positron emission tomography; PSP, progressive supranuclear palsy; QSM, quantitative susceptibility mapping; RBD, rapid eye movement sleep behavior disorder; SAA, seed amplification assay; SNpc, substantia nigra pars compacta; SPECT, single-photon emission computed tomography; TSE, turbo spin echo.

## 2 Literature search strategy

This article is a narrative review. PubMed and the Web of Science Core Collection were last searched on June 30, 2026, for publications dated from January 1, 2006, to June 30, 2026. The main PubMed search query was as follows: (“Parkinson Disease”[Mesh] OR “Parkinson disease”[Title/Abstract] OR “Parkinson’s disease”[Title/Abstract] OR parkinsonism[Title/Abstract]) AND (“neuromelanin-sensitive MRI”[Title/Abstract] OR “neuromelanin-sensitive magnetic resonance imaging”[Title/Abstract] OR “neuromelanin MRI”[Title/Abstract] OR “neuromelanin imaging”[Title/Abstract] OR “NM-MRI”[Title/Abstract]). The main Web of Science query was as follows: TS = ((“Parkinson disease” OR “Parkinson’s disease” OR parkinsonism) AND (“neuromelanin-sensitive MRI” OR “neuromelanin-sensitive magnetic resonance imaging” OR “neuromelanin MRI” OR “neuromelanin imaging” OR “NM-MRI”)). Supplementary searches combined NM-MRI-related terms with the following topic terms: “differential diagnosis,” “prodromal,” “rapid eye movement sleep behavior disorder,” “quantitative susceptibility mapping,” “diffusion MRI,” “functional MRI,” “artificial intelligence,” “machine learning,” “radiomics,” and “alpha-synuclein seed amplification assay.”

The verifiable PubMed export comprised 170 records, 59 of which were retained as core candidates for full-text review. Web of Science was used to identify additional studies and for citation tracking; however, a separately verifiable count after cross-database deduplication was not retained. Following full-text review, Supplementary Table S1 included 37 original clinical studies reporting numerical diagnostic or classification performance and separately listed two meta-analyses of diagnostic performance. Study selection and data extraction were performed by a single author without independent duplicate screening. For inclusion among the “representative studies” in Supplementary Table S1, priority was given to studies with a clearly defined clinical task, extractable information on sample composition and NM-MRI measures, numerical performance metrics such as AUC, sensitivity, specificity, or accuracy, or a clearly specified validation design. Seminal early studies were also retained. This process should not be interpreted as an exhaustive study-selection process equivalent to that of a systematic review.

The original clinical studies summarized in Supplementary Table S1 were screened using the PICOS framework shown in Table 1. Studies were required to evaluate NM-MRI for diagnostic support in PD, differential diagnosis, prodromal assessment, or a related classification task and to report at least one extractable numerical diagnostic or classification outcome. Studies reporting only between-group differences, correlations, or qualitative findings were not included in Supplementary Table S1. When cohorts overlapped, the report with the most complete diagnostic performance data was retained, and companion articles were used only to supplement missing information. When a single study addressed different classification tasks, measures, or models, these were labeled separately, and their performance estimates were not pooled or interpreted as a single task. If data reported in a table differed from those in the abstract or secondary sources, the original full text and final analytical sample took precedence. Mechanistic studies, imaging-pathology correlation studies, systematic reviews, and other clinical studies discussed in the main text but not included among the diagnostic performance studies in Supplementary Table S1 were selected through a narrative process according to their relevance to the technical basis, biological mechanisms, and clinical applications of NM-MRI. They were not restricted by the diagnostic performance criteria in Table 1, and this process did not constitute an exhaustive systematic review search and selection process.

**Table 1:** Eligibility criteria for diagnostic-performance studies of NM-MRI in Parkinson’s disease.

PICOS domain (adapted for diagnostic-accuracy studies)	Inclusion criteria	Exclusion criteria
Population/target condition	Human participants with clinically diagnosed Parkinson’s disease (PD), at-risk prodromal syndromes (e.g., iRBD), clinically relevant differential diagnoses (e.g., ET, PSP, MSA-P), or predefined PD phenotypes/subtypes. The target condition or classification task must be clearly defined.	Animal studies, *in vitro* studies, or studies based exclusively on postmortem data; single case reports; studies without a clearly defined target condition or classification task.
Index test	Dedicated NM-MRI or an explicitly identified NM-sensitive image derived from another MRI acquisition must be evaluated as the index test. NM-MRI-only performance must be extractable, or NM-MRI must be a prespecified feature in a clearly labeled multimodal or AI-based model.	Studies without NM-MRI; studies in which NM-MRI is used only to delineate an ROI while the evaluated diagnostic marker is exclusively DTI, QSM, SWI, T2*, or another non-NM measure.
Reference standard / comparator	An explicit and clinically reasonable reference standard—for example, accepted diagnostic criteria, expert clinical diagnosis, or an appropriate imaging reference—must be applied to the study participants, with healthy controls or disease-control cohorts included as appropriate.	No reference standard or comparator; unclear case definition; uncontrolled single-case evidence.
Diagnostic outcome	At least one extractable numerical diagnostic/classification result: AUC, sensitivity, specificity, accuracy, PPV, NPV, likelihood ratios, or data sufficient to reconstruct a 2 × 2 table. For AI studies, performance must be reported for a held-out test or validation set.	Only group differences, correlations, *p* values, technical repeatability, or qualitative statements such as ‘good diagnostic value’ without any extractable numerical performance result.
Study design / report type	Peer-reviewed, full-text human original diagnostic-accuracy studies (case–control, cross-sectional, or cohort). Technical or AI studies are eligible only when numerical diagnostic performance is reported. Diagnostic-accuracy systematic reviews/meta-analyses may be displayed in a separate table section. English-language publications.	Narrative reviews, editorials/letters, case reports, conference abstracts, unreviewed preprints, non-English publications, and technical-validation studies without diagnostic-performance data.
Overlapping cohorts	When multiple reports use an overlapping cohort, retain the report with the most complete and directly extractable diagnostic-performance data; companion reports may be used only to clarify missing details.	Duplicate counting of the same or substantially overlapping cohort when a more complete diagnostic report is available.

The PICOS framework was adapted for diagnostic-accuracy studies: the conventional intervention and comparator domains were operationalized as the index test and the reference standard/comparator, respectively. Studies used only for technical background, biological mechanisms, or imaging-pathology interpretation were not subjected to these clinical diagnostic-performance criteria. AI, artificial intelligence; AUC, area under the receiver operating characteristic curve; DTI, diffusion tensor imaging; ET, essential tremor; HC, healthy control(s); iRBD, isolated rapid eye movement sleep behavior disorder; LC, locus coeruleus; LR, likelihood ratio; MRI, magnetic resonance imaging; MSA-P, multiple system atrophy, parkinsonian type; NM, neuromelanin; NM-MRI, neuromelanin-sensitive magnetic resonance imaging; NPV, negative predictive value; PD, Parkinson’s disease; PICOS, population, index test, comparator/reference standard, outcomes, and study design; PPV, positive predictive value; PSP, progressive supranuclear palsy; QSM, quantitative susceptibility mapping; SNpc, substantia nigra pars compacta; SWI, susceptibility-weighted imaging.

## 3 Technical basis and recent advances in NM-MRI

NM-MRI contrast is not a direct quantitative measure of neuromelanin and may arise from multiple factors, including the paramagnetic properties of neuromelanin-iron complexes, tissue water content, and magnetization transfer (MT) effects (2, 4). Early NM-MRI studies generally used two-dimensional T1-weighted fast spin-echo or turbo spin-echo sequences, in which repeated radiofrequency pulses can generate an endogenous MT effect. More recent three-dimensional gradient-echo approaches can enhance neuromelanin-related image contrast in the SNpc and LC through explicit MT preparation pulses (5, 13). Although early two-dimensional techniques can depict the SNpc and LC, they are generally limited by relatively long acquisition times and restricted brain coverage (13).

Recent technical advances have improved the practicality and robustness of NM-MRI. One notable innovation is the sandwich spatial saturation NM-MRI method, in which spatial saturation radiofrequency pulses are applied above and below the imaging volume to enhance MT weighting and reduce asymmetric MT effects (14). Compared with conventional MT-prepared sequences, this method produced more consistent neuromelanin-related image contrast in a small multivendor experiment, suggesting that it may improve consistency across centers; however, validation in larger clinical samples is still required (14). Another emerging method is Proton-density Enhanced Neuromelanin Contrast in Low flip-angle gradient echo (PENCIL) imaging, which enables relatively rapid whole-brain coverage and improves the contrast-to-noise ratio. Its performance in distinguishing PD from healthy controls is comparable to that of standard magnetization transfer contrast methods (15). PENCIL can be implemented within the strategically acquired gradient echo (STAGE) framework, which has also been used to obtain neuromelanin-, nigrosome 1-, and iron-related measures in a single protocol (15, 16). These methods may improve the accessibility of NM-MRI and expand its capacity for multiparametric assessment.

## 4 Evidence synthesis: diagnostic utility of NM-MRI in PD

A relatively large number of studies have examined the ability of NM-MRI to distinguish patients with PD from healthy controls. A meta-analysis of 12 studies, comprising 403 patients with PD and 298 healthy controls, reported a pooled sensitivity of 89% (95% confidence interval [CI], 86–92%) and a pooled specificity of 83% (95% CI, 76–88%) (17). Subgroup and meta-regression analyses suggested that disease duration, measurement type, slice thickness, and segmentation method may influence diagnostic performance across studies. Slice thickness greater than 2 mm, volumetric measurement, and semi-automated or automated segmentation were associated with higher performance; however, these observational subgroup findings cannot be interpreted as causal evidence for optimal acquisition parameters (17). Another meta-analysis reported pooled sensitivity and specificity values of 82% (95% CI, 74–87%) and 82% (95% CI, 73–89%), respectively, and identified SNpc volume as a potentially promising measure (19). In a multicenter study, NM-MRI contrast and volume measures distinguished early PD from controls with accuracies of 85 and 83%, respectively (8). The sample composition, specific classification tasks, NM-MRI measures, validation methods, and diagnostic or classification performance of representative studies (20, 22, 28, 29, 33, 34, 37, 38, 48, 50, 51) are summarized in Supplementary Table S1.

The spatial pattern of reduced neuromelanin-related image contrast within the SNpc appears to follow a characteristic trajectory that may reflect regional structural changes associated with PD. Contrast changes first emerge in the posterolateral motor region of the SNpc and extend medially as the disease progresses (9). This spatiotemporal pattern is broadly consistent with postmortem histological findings and supports further investigation of NM-MRI for disease staging. Some studies have reported associations between NM-MRI-derived measures and clinical ratings of motor impairment, such as the Unified Parkinson’s Disease Rating Scale, as well as cognitive or affective symptoms, suggesting that these measures may reflect clinically meaningful neurodegenerative changes (9, 31, 45, 46). Reduced SNpc and LC volumes have also been replicated in two independent cohorts (52). Associations between NM-MRI measures and striatal dopaminergic function on DAT PET or SPECT further support its potential as a complementary marker of nigrostriatal integrity (53, 54). In early PD cohorts, ventral SNpc neuromelanin-related imaging measures correlated with striatal DAT binding in the corresponding anatomical projection territories, suggesting that nigrostriatal degeneration may exhibit a degree of topographic organization (55).

## 5 Evidence synthesis: NM-MRI in the differential diagnosis of parkinsonian syndromes

Distinguishing PD from atypical parkinsonian syndromes (APS) and other tremor disorders, particularly in the early stages of disease, remains an important clinical challenge (6). NM-MRI may provide complementary information through patterns of neuromelanin-related image contrast across different nuclei and subregions. In a cohort with early parkinsonism, lateral substantia nigra pars compacta (SNpc) contrast was lower in patients with PD and multiple system atrophy, parkinsonian type (MSA-P) than in those with progressive supranuclear palsy (PSP) and healthy controls, whereas LC contrast was lower in PD than in MSA-P, PSP, and healthy controls. The sensitivity and specificity for distinguishing PD from MSA-P were 60 and 90%, respectively, indicating group-level differences but insufficient reliability of a single measure for individual differential diagnosis (49). A more recent multimodal study showed that combining SNpc volume, red nucleus susceptibility, and the midbrain-to-pons ratio improved the differentiation of PSP from PD, although the findings were derived from a single cohort (24, 44).

NM-MRI may also aid in distinguishing PD from essential tremor (ET), whose clinical manifestations may overlap with those of PD. Compared with patients with ET, those with tremor-dominant PD showed lower neuromelanin-related volumes and contrast-to-noise ratios in the SN and LC; the combined model achieved an AUC of 0.92 (35). Other single-center studies have also reported that SN area, contrast, or visual ratings may help differentiate PD from ET, although sensitivity and specificity have varied across studies (23, 30, 42, 47). For drug-induced or vascular parkinsonism, review-level evidence suggests that neuromelanin-related SNpc signal may be relatively preserved. Direct comparative studies are limited, however, and the absence of signal reduction should not be used as a definitive criterion to exclude PD (6). DAT imaging remains an important clinical tool for evaluating nigrostriatal dopaminergic function, whereas NM-MRI is better positioned as a non-ionizing adjunct that can be incorporated into routine MRI protocols (6, 7).

Overall, several single-center studies support the use of NM-MRI in differentiating PD from essential tremor. Evidence is more limited for drug-induced and vascular parkinsonism and for distinguishing PD from PSP or MSA-P, and is constrained by overlapping imaging features across diseases, limited sample sizes, and a lack of independent external validation (6, 24, 35, 42, 47, 49).

## 6 Evidence synthesis: NM-MRI in prodromal assessment, staging, and longitudinal monitoring

Although dopamine transporter (DAT) imaging remains an important clinical method for assessing the function of striatal dopaminergic nerve terminals, NM-MRI generates neuromelanin-related image contrast in the substantia nigra pars compacta (SNpc) without ionizing radiation and can indirectly reflect its structural integrity, making it a potential complement to DAT imaging (6, 7). SNpc volume measured with NM-MRI and SNpc free water (FW) derived from diffusion MRI provide information about nigral structural integrity and tissue microstructure, respectively. A case–control study found reduced SNpc volume and increased FW in patients with PD, whereas these measures in individuals with scans without evidence of dopaminergic deficit (SWEDD) were similar to those in healthy controls. Because the study neither developed nor validated a combined diagnostic model incorporating both measures, these findings do not support their use as an alternative diagnostic strategy when DAT imaging is unavailable or inconclusive (56). In addition, an exploratory study in advanced PD suggested that three-dimensional NM-MRI and DAT SPECT may provide information across different domains. LC volume was associated with the severity of preoperative sleep disturbance and with the mobility dimension of the Parkinson’s Disease Questionnaire-39 1 year after deep brain stimulation, whereas the DAT-specific binding ratio was associated with cognitive processing speed and visuospatial ability. After correction for multiple comparisons, substantia nigra volume was not significantly associated with clinical ratings (57). These findings provide preliminary support for the complementary value of the two imaging methods, but require validation in larger samples.

The potential role of NM-MRI in assessing prodromal PD has also attracted attention. Early studies of isolated rapid eye movement sleep behavior disorder (iRBD) mainly reported reduced signal in the LC/subcoeruleus complex (11, 12), whereas more recent work has extended the evidence to the SNpc. A cross-sectional Parkinson’s Progression Markers Initiative study included 50 controls, 90 nonmanifesting genetic variant carriers, 217 prodromal participants with hyposmia and DAT deficit, 76 participants with RBD, 194 patients with newly diagnosed PD, and 26 patients with moderate PD. Nigral volume was lower in both the hyposmia and RBD groups than in controls, whereas no significant differences were observed between nonmanifesting *LRRK2* or *GBA1* carriers and controls. These findings suggest that NM-MRI changes are detectable in some, but not all, prodromal or high-risk populations (70).

Another longitudinal study integrating animal, histological, and human data included 52 healthy volunteers, 36 participants with iRBD (29 who did not phenoconvert and 7 who phenoconverted during follow-up), and 145 patients with early PD. The study identified a biphasic trajectory in which the NM-MRI signal initially increased with neuromelanin accumulation and subsequently decreased with neurodegeneration. Signal reduction was observed in both iRBD phenoconverters and patients with early PD; however, the number of phenoconverters was small and some between-group comparisons of slopes did not reach statistical significance, so the findings should be regarded as exploratory longitudinal evidence (69). In addition, a previous cohort including participants with iRBD, patients with PD, and healthy controls used SNpc-related morphological and signal changes to construct a disease-timeline model. The model estimated that the decline in relevant imaging measures may begin approximately 5.3 years before clinical diagnosis and that SNpc-related volume may already be reduced by approximately 23.1% relative to healthy controls at diagnosis (9). These estimates represent group-level model inferences and cannot provide deterministic predictions of disease onset for individual patients. Neuromelanin-related imaging patterns in *LRRK2*-associated PD or asymptomatic *LRRK2* variant carriers may also differ from those in idiopathic PD, suggesting research potential for stratifying genetic subtypes (58, 59, 70).

As the disease progresses, the potential application of NM-MRI for longitudinal monitoring has received increasing attention. Longitudinal cohort studies suggest that NM-MRI can detect reductions in neuromelanin-related SNpc image contrast and volume, and that these imaging changes are associated with motor decline (8, 10). A prospective study of 140 patients with PD demonstrated a significant reduction in SNpc volume over 2 years, with a greater decline among those showing more rapid clinical progression (10). NM-MRI may therefore represent a candidate objective outcome measure for disease-modifying clinical trials and may help optimize sample-size requirements and follow-up intervals. Nevertheless, its reliability as a clinical trial outcome and its sensitivity to treatment response require further validation (8).

Thus, NM-MRI has research value for detecting prodromal or longitudinal changes at the group level, but independently validated thresholds for individual risk are not yet available, and adequate sensitivity to disease-modifying treatment effects and specificity for disease-related change have not been demonstrated (8–12).

## 7 Evidence from multimodal imaging and artificial intelligence studies

The pathology of Parkinson’s disease (PD) encompasses neuromelanin loss, iron deposition, microstructural injury, and dopaminergic dysfunction, and no single imaging measure can fully characterize nigral degeneration. Integrating neuromelanin-sensitive magnetic resonance imaging (NM-MRI) with quantitative susceptibility mapping (QSM), diffusion MRI, and other imaging methods may therefore provide complementary information across distinct pathological dimensions (21, 32, 39, 43, 53, 60).

For example, Chen et al. (24) prospectively enrolled 73 patients with PD, 32 patients with progressive supranuclear palsy (PSP), and 36 healthy controls. The midbrain-to-pons ratio (MPR) was measured on midsagittal T1-weighted images, and NM-MRI and QSM were acquired simultaneously using a single three-dimensional multi-echo magnetization transfer contrast gradient-echo sequence to measure substantia nigra pars compacta (SNpc) volume and red nucleus susceptibility. The AUCs for distinguishing PSP from PD using MPR, SNpc volume, and red nucleus susceptibility alone were 0.875, 0.883, and 0.892, respectively. Combining SNpc volume and red nucleus susceptibility increased the AUC to 0.928, and combining all three measures yielded an AUC of 0.939 (24). However, diagnostic performance was evaluated within the same study cohort without independent external validation, and the cross-center generalizability of these findings remains to be established.

Jin et al. (42) enrolled 68 untreated patients with newly diagnosed PD, 25 untreated patients with essential tremor, and 34 healthy controls, and jointly evaluated the neuromelanin-related signal on NM-MRI and the nigrosome 1 signal on QSM. For differentiating PD from essential tremor, NM-MRI alone achieved a sensitivity of 88.2% and a specificity of 80.0%, whereas nigrosome 1 imaging alone achieved a sensitivity of 79.4% and a specificity of 92.0%. Combining the two measures yielded an AUC of 0.935, a sensitivity of 85.3%, and a specificity of 92.0% (42). These findings indicate that combining complementary imaging measures can improve differentiation within the same cohort. In the absence of independent external validation, however, they do not establish cross-center robustness or generalizability.

NM-MRI can also be combined with diffusion MRI measures. Pyatigorskaya et al. (43) prospectively enrolled 36 patients with PD and 20 healthy controls. The 3 T MRI protocol included three-dimensional T2-weighted imaging, T1-weighted NM-MRI, diffusion tensor imaging (DTI), and quantitative effective transverse relaxation rate (R2*) mapping. High-resolution T2*-weighted images were also acquired at 7 T to assess dorsolateral nigral hyperintensity. The best combined model, comprising SNpc volume and neuromelanin-related signal intensity on NM-MRI together with fractional anisotropy (FA), achieved a diagnostic accuracy of 0.93, sensitivity of 0.92, specificity of 0.95, and an AUC of 0.96 (43). Because this model did not include R2* or 7 T T2* measures, the findings primarily support complementarity between NM-MRI and diffusion measures and do not demonstrate a direct performance gain from iron-sensitive measures. The study also had a small sample and lacked an independent external validation cohort.

Current applications of artificial intelligence to NM-MRI have focused primarily on localizing the brainstem or SNpc, extracting imaging features, and classifying disease (36). A small number of studies have begun to explore the fusion of radiomic and deep-learning features. However, studies integrating genuinely distinct imaging modalities, such as NM-MRI and QSM, remain scarce, as do studies incorporating fully independent external validation (26, 27, 41).

Shinde et al. (41) included 45 patients with PD, 20 patients with atypical parkinsonian syndromes, and 35 healthy controls. For the classification of PD versus healthy controls, 30 patients with PD and 25 healthy controls were used for model training and five-fold cross-validation, and an additional 15 patients with PD and 10 healthy controls served as the test set. The convolutional neural network learned SNpc-related spatial patterns directly from NM-MRI images and achieved a test accuracy of 80.0%, compared with 56.5% for the contrast-ratio method and 60.3% for a conventional radiomics classifier. Test accuracy for differentiating PD from atypical parkinsonian syndromes was 85.7% (41). However, both the training and test sets were drawn from the same center, and the test sample was small; overfitting and center-specific bias therefore remain possible.

Chen et al. (27) enrolled 73 patients with early PD and 65 healthy controls. The 138 participants were divided into a development set of 111 participants and a held-out internal test set of 27 participants; an additional 24 participants from another center were included for external validation. The input was a neuromelanin-sensitive short-echo-time magnitude image (setMag) reconstructed from a multi-echo QSM acquisition (40). The investigators first used YOLO-v5 to localize the brainstem and then extracted deep-learning features with a modified LeNet. They also extracted 1,781 radiomic features from the SNpc, retaining 10 after screening based on intraclass correlation coefficients and the least absolute shrinkage and selection operator (LASSO). Classification models were developed using radiomic features, deep-learning features, and a fusion of the two feature classes. The “hybrid model” therefore represented feature-level fusion of radiomic and deep-learning features rather than multimodal input of separate QSM and NM-MRI images. Five-fold cross-validation was used during model development, and the support vector machine based on fused features achieved accuracies of 96.3% in the held-out internal test set and 95.8% in the external validation set (27). Although external validation was performed, the external cohort comprised only 24 participants, and cross-center generalizability requires confirmation in larger, multicenter, multivendor datasets.

Welton et al. (26) performed independent external validation of two existing deep-learning models based on susceptibility map-weighted imaging (SMWI) in 82 patients with PD and 107 healthy controls. They also evaluated an iron-neuromelanin composite measure constructed from manually segmented QSM and NM-MRI measurements. The two deep-learning models were designed to detect morphological abnormalities and volume changes in nigrosome 1, whereas the QSM-NMS composite was derived from manually segmented quantitative measurements and was not a deep-learning model. The AUCs of the nigrosome 1 morphology model, volume model, and QSM-NMS composite measure were 0.92, 0.90, and 0.94, respectively, compared with 0.98 for an experienced neuroradiologist (26). All 18 participants misclassified by the nigrosome 1 morphology model were healthy controls; therefore, all misclassifications were false positives. Potential explanations for these false-positive classifications included left–right asymmetry, slice-reconstruction effects, pulsation artifacts, and a thin appearance of nigrosome 1 (26). These findings support the potential of artificial intelligence as a diagnostic aid, although its AUC point estimate was lower than that of the experienced neuroradiologist. Because the study did not report a formal statistical comparison of the AUCs, it would be inappropriate to conclude definitively that artificial intelligence either outperformed or underperformed the specialist.

Despite the high classification performance reported in these studies, current artificial intelligence studies share several limitations. First, the number of independent participants in most studies remains small relative to the number of radiomic features or the complexity of the deep-learning models, creating a substantial risk of overfitting. Data augmentation can increase the number of training images but not the number of independent participants, and internal random splits or cross-validation cannot replace genuinely independent external validation (27, 41). In the broader literature on prodromal PD detection, a scoping review of nine studies comprising 567 patients with prodromal PD and 35,643 controls reported sensitivities ranging from 43 to 84% and specificities of up to 96% across imaging and data-driven methods. Study heterogeneity and inadequate validation remained major barriers to clinical translation (61). Importantly, the review covered diverse neuroimaging, electrocardiographic, accelerometer, speech analysis, and machine-learning methods; these performance ranges cannot be interpreted as the overall diagnostic performance of artificial intelligence models based on NM-MRI.

Second, differences in scanners and acquisition protocols may limit the cross-center generalizability of quantitative NM-MRI measures and related artificial intelligence models. NM-MRI signals are susceptible to technical factors including scanner vendor, radiofrequency coil, sequence parameters, slice thickness, interslice gap, and reconstruction method (14, 62). The sandwich spatial saturation NM-MRI method proposed by Ji et al. (14) reduced cross-device variability in neuromelanin-related contrast in a small multivendor experiment, suggesting that it may improve the consistency of multicenter NM-MRI acquisition; validation in larger samples and clinical populations is nevertheless required. Hirose et al. (62) compared NM-MRI results obtained within 3 months using two different MRI protocols in 54 patients with PD. Neither the SNpc contrast ratio (*r* = 0.233, *p* = 0.091) nor the locus coeruleus (LC) contrast ratio (Spearman’s *ρ* = 0.180, *p* = 0.192) was significantly correlated between protocols, and SNpc area showed only a weak correlation (Spearman’s ρ = 0.403, *p* = 0.003) (62). The investigators also observed systematic measurement bias between protocols, indicating that quantitative NM-MRI measures obtained using different equipment and acquisition protocols are not directly interchangeable. Consequently, model performance may decline because of technical domain shift when training and validation datasets are acquired on different scanners or at different centers. Conversely, if disease labels are strongly correlated with imaging site, a model may learn scanner- or site-specific features rather than PD-related pathology.

Third, the interpretability of models and the transparency and stability of complex interactions among imaging features remain limited. Class activation maps and saliency maps can indicate the image regions or features on which a model focuses (27, 41), but they do not establish a causal relationship between these features and disease or guarantee stable explanations across populations, scanners, and acquisition protocols. Left–right asymmetry, slice reconstruction, pulsation artifacts, and a thin nigrosome 1 may also influence model decisions (26). In feature-fusion models, nonlinear interactions between radiomic and deep-learning features are often difficult to translate into clinically intelligible and reproducible decision rules.

Finally, a substantial gap remains between model performance in research settings and clinical deployment. Most studies compare patients with established PD with healthy controls, whereas real-world clinical populations encompass a more complex spectrum, including prodromal PD, diagnostically uncertain cases, essential tremor, drug-induced parkinsonism, PSP, and multiple system atrophy (26, 61). A high AUC or accuracy in a study cohort of carefully selected cases and healthy controls does not necessarily indicate good probability calibration, clinical net benefit, or an acceptable false-positive rate. Practical deployment may also be affected by the need for dedicated multi-echo sequences, additional image post-processing, artifact detection, computational time, integration into clinical workstations, and regulatory approval (26).

Future studies should use large, multicenter, multivendor datasets; prespecify training, validation, and test splits at the participant level; and incorporate nested cross-validation, leave-one-center-out validation, and genuinely independent external validation. In addition to AUC and accuracy, investigators should fully report sensitivity, specificity, positive and negative predictive values, F1 score, confidence intervals, probability calibration, and decision-curve analysis, and should compare radiologists alone, artificial intelligence alone, and radiologists assisted by artificial intelligence. Multimodal studies should clearly distinguish image-level, feature-level, and decision-level fusion and assess the effects of missing modalities, changes in acquisition protocol, and imaging artifacts on model performance. Artificial intelligence and multimodal NM-MRI models can progress from research tools to deployable clinical decision-support systems only after acquisition has been standardized and cross-center generalizability, prospective clinical validity, model interpretability, and integration into clinical workflows have been demonstrated.

## 8 Discussion: clinical positioning, limitations of the evidence, and future directions

Taken together, a relatively large body of research has evaluated NM-MRI for distinguishing patients with established PD from healthy controls, although the highest diagnostic performance estimates have arisen mainly from highly selected case–control designs (17, 18). Evidence for differential diagnosis involving PSP, essential tremor, and related conditions is increasing, but most findings still derive from single-center cohorts (6, 24, 35, 49). Prodromal detection, individualized prediction of progression, and use as a clinical trial outcome remain exploratory (8–12, 69, 70). Although multimodal and artificial intelligence models have achieved high AUCs or accuracies, limited sample sizes, inconsistent validation strategies, and inadequate independent external validation mean that these results cannot yet be taken as evidence of utility in routine clinical practice (24, 26, 27, 41, 61). To make the assessment of the maturity of the evidence transparent and verifiable, Table 2 summarizes the types of evidence, current level of readiness, and key gaps for each clinical application. As illustrated in Figure 1, the principal value of NM-MRI currently lies in research and in its potential use as an adjunct to clinical assessment.

**Table 2:** Maturity of evidence and key research gaps across clinical applications of neuromelanin-sensitive MRI.

Clinical application	Current evidence	Principal evidence type	Current level of readiness	Key research gaps
PD versus healthy controls	Relatively numerous studies are available, but most have used highly selected case–control designs (18, 19).	Case–control studies, systematic reviews, and meta-analyses	Research-stage diagnostic adjunct	Prospective cohorts with consecutive enrollment; standardized acquisition protocols and analytical thresholds; demonstration of incremental clinical value
Differential diagnosis	Several single-center studies have compared PD with ET; evidence for PD versus PSP, MSA-P, and related parkinsonian syndromes remains more limited (7, 24–26).	Single-center comparative studies and a small number of multimodal studies	Exploratory aid to differential diagnosis	External validation across the full clinical spectrum; cross-center reproducibility; comparison with existing diagnostic pathways
Prodromal detection	iRBD, hyposmia, and genetically defined at-risk populations have been studied; findings are inconsistent across populations, and longitudinal phenoconverter samples remain very small (5, 10, 12, 13, 32).	iRBD cohorts, a cross-sectional PPMI cohort, and small longitudinal cohorts	Exploratory candidate biomarker	Larger phenoconverter samples; validated individual-level risk thresholds; long-term predictive performance; independent validation
Monitoring disease progression	Single-center and multicenter longitudinal studies suggest that SNpc-related contrast or volume declines over time (9, 11).	Observational longitudinal cohorts	Candidate biomarker for monitoring progression	Test–retest reliability; minimum detectable change; individual-level interpretability; cross-protocol consistency
Clinical trial outcome measure	Only observational longitudinal evidence is available; validation in therapeutic intervention trials is lacking (9, 11).	Observational longitudinal studies; no validation in interventional trials	Unvalidated candidate outcome measure	Sensitivity to treatment effects; stability of effect-size estimates; sample-size estimation; qualification as a clinical trial endpoint
Routine clinical practice	Prospective evidence of clinical utility, implementation, and cost-effectiveness is lacking (7).	Reviews and a limited number of clinical studies	Not ready for routine clinical use	Standardization; external validation; incremental clinical utility; cost-effectiveness; workflow integration

This table presents a narrative synthesis of the principal evidence types, current level of readiness, and key research gaps across clinical applications of neuromelanin-sensitive MRI (NM-MRI), based on the studies included in this review. Readiness was assessed qualitatively according to the number and design of available studies, sample representativeness, multicenter and longitudinal evidence, independent external validation, and evidence of clinical utility. The table does not constitute a formal evidence grade generated using GRADE or another structured evidence-rating framework. The readiness descriptors indicate only the current stage of research and clinical translation; they do not imply that NM-MRI is established for stand-alone diagnosis, individual-level disease prediction, monitoring treatment response, or routine clinical decision-making. Citation numbers correspond to those in the main reference list.

ET, essential tremor; iRBD, isolated rapid eye movement sleep behavior disorder; MSA-P, multiple system atrophy, parkinsonian type; NM-MRI, neuromelanin-sensitive magnetic resonance imaging; PD, Parkinson’s disease; PPMI, Parkinson’s Progression Markers Initiative; PSP, progressive supranuclear palsy; SNpc, substantia nigra pars compacta.

Despite its promise, NM-MRI faces several challenges in the transition to widespread clinical use. First, acquisition protocols and analytical methods are not standardized across centers, resulting in substantial heterogeneity in diagnostic performance (17, 63). Harmonizing acquisition parameters for approaches such as sandwich spatial saturation NM-MRI may improve reproducibility across MRI systems from different vendors (14), while automated segmentation tools and template-based analytical pipelines may reduce operator dependence (8, 19, 25). Second, high-resolution NM-MRI requires relatively long scan times, whereas PENCIL, which enables rapid whole-brain coverage, may improve clinical feasibility (15). Finally, the biological and physical sources of the NM-MRI signal remain debated. T1 shortening related to neuromelanin and neuromelanin–iron complexes, differences in tissue proton density and water content, and MT effects may all contribute, and their relative importance across sequences and disease stages remains uncertain (2, 4, 69). No single mechanism should therefore be regarded as an established dominant explanation.

Multimodal studies further suggest that NM-MRI can be combined with neurophysiological and functional imaging to relate the integrity of brainstem nuclei to brain-network dysfunction. Wiesman et al. (64) combined NM-MRI with resting-state magnetoencephalography (MEG) and found that lower LC neuromelanin-related measures in patients with PD were associated with abnormal alpha-band activity in frontomotor cortical regions and impaired attention. Lower SN neuromelanin-related measures were associated with reduced beta activity in the left lateral somatosensorimotor region, and the spatial distribution of the beta-SN association corresponded to the regional map relating beta activity to axial motor symptoms. This finding represents spatial colocalization of group-level maps and does not establish a direct participant-level association between SN neuromelanin measures and axial symptoms. Using NM-MRI together with functional MRI during a stop-signal task, Xu et al. (65) found that reduced LC integrity in PD was accompanied by a longer stop-signal reaction time and attenuated stopping-related activity in the right inferior frontal gyrus, pre-supplementary motor area (pre-SMA), and right caudate nucleus. In healthy controls, LC integrity was associated with stopping-related activity in the right inferior frontal gyrus and subthalamic nucleus; this association was no longer significant in patients with PD, suggesting that LC degeneration may compromise inhibitory control mediated by the fronto-subthalamic “hyperdirect” pathway (65).

Within the broader biomarker landscape, NM-MRI, fluid biomarkers, and peripheral tissue markers capture different biological dimensions. *α*-synuclein seed amplification assays (SAAs) are used primarily to detect seeding-competent pathological α-synuclein, whereas NM-MRI provides regional information related to the structural integrity of catecholaminergic nuclei such as the SNpc and LC. The two are therefore better regarded as candidate biomarkers reflecting distinct pathological dimensions rather than as interchangeable tests (3). Mangone et al. (67) found no significant association between α-synuclein deposition in labial minor salivary gland tissue and SNpc injury on NM-MRI. However, the study had a limited sample size and used immunolabeling in peripheral tissue rather than a fluid biomarker or seed amplification assay. It therefore cannot be used to infer the absence of an association between SAA and NM-MRI or to demonstrate the incremental diagnostic or prognostic value of combining them. Complementarity among biomarkers must be evaluated in prespecified joint models and prospective studies.

Future research should focus on validating NM-MRI with standardized protocols in large, multicenter longitudinal cohorts and on evaluating the incremental value of combining it with other imaging, fluid, and tissue biomarkers (3). Applying machine-learning methods to multimodal imaging data may improve the identification and risk stratification of prodromal PD, although their value for individualized prediction of disease progression requires longitudinal validation (61). As disease-modifying therapies for PD advance through clinical development, biomarkers capable of detecting disease-related changes early and monitoring treatment response are becoming increasingly important. NM-MRI and multimodal approaches may provide complementary information in this setting.

This review also has methodological limitations. The literature search covered only PubMed and the Web of Science Core Collection, did not include Embase or gray literature, and was restricted to English-language publications, potentially introducing database-related selection bias, publication bias, and language bias. The available PubMed export permits verification of the initial retrieval count and the number of core candidates, but independent Web of Science counts, a complete cross-database deduplication log, and article-level reasons for exclusion were not retained. Screening and data extraction were not performed independently by two reviewers. As a narrative review, this work did not include protocol registration, PRISMA flow reporting, or a formal risk-of-bias assessment. Thus, although Supplementary Table S1 summarizes representative diagnostic performance studies according to the PICOS criteria in Table 1 and presents meta-analyses separately, it should not be interpreted as an exhaustive list of all eligible studies. The conclusions should be interpreted in the context of study heterogeneity and the methodological limitations described above.

## 9 Conclusions and future perspectives

In conclusion, neuromelanin-sensitive magnetic resonance imaging (NM-MRI) is a promising noninvasive imaging biomarker. It generates neuromelanin-related image contrast in the substantia nigra pars compacta (SNpc) and locus coeruleus (LC) and can indirectly reflect the structural integrity of these catecholaminergic nuclei (4, 13). Current evidence suggests that NM-MRI may have complementary value for distinguishing patients with PD from healthy controls, supporting the differential diagnosis of selected atypical parkinsonian syndromes and essential tremor, monitoring disease progression, and assessing high-risk individuals in the prodromal phase (6, 8, 10, 35, 66). However, the available evidence still comes largely from single-center or case–control studies with limited sample sizes, and clinical utility requires further evaluation in prospective, multicenter longitudinal studies.

Overall, NM-MRI has potential complementary value within the PD biomarker landscape. As biomarker-based frameworks for disease assessment continue to evolve and acquisition and analytical methods become increasingly standardized, NM-MRI may become a component of multimodal assessment. When combined with other neuroimaging measures and fluid biomarkers, it may provide complementary information for early detection, disease stratification, progression monitoring, and assessment of treatment response (3, 68). Before adoption in routine clinical practice, NM-MRI requires further evaluation in large, prospective, multicenter longitudinal studies and independent external validation.
