# Supplementary material for: Neuromelanin-sensitive magnetic resonance imaging in the diagnosis and assessment of Parkinson’s disease
Source: Front Neurol. 2026 Oct 5;17:1908455. doi: 10.3389/fneur.2026.1908455 (PMC13678384; doi:10.3389/fneur.2026.1908455)
Supplement: Supplementary file 1 [file Table_1.DOCX]

**TABLE 2 | Diagnostic and classification performance of NM-MRI in Parkinson's disease and related cohorts**

| **Study** | **Year** | **Patient group n** | **Comparator n** | **Field** | **ROI** | **NM-MRI metric** | **Sensitivity (%)** | **Specificity (%)** | **AUC / accuracy** |
| --- | --- | --- | --- | --- | --- | --- | --- | --- | --- |
| **Original studies** | |  |  |  |  |  |  |  |  |
| **Silovs A et al. [19]** | 2026 | 25 | 25 (HC) | 3T | SN | Semi-automated volume | 68.4 | 74.1 | 0.700 |
| **Imokawa T et al. [20]** | 2026 | 24 | 24 (HC) | NR | SNc/LC | NM-only: SPIR signal ratio | NR | NR | Accuracy 87.5% |
| **Yu Q et al. [21]** | 2026 | 72 | 34 (PD mimics) | 3T | Midbrain | NM-MRI component within a multimodal protocol: neuromelanin measure | NR | NR | 0.64 |
| **Fushimi Y et al. [16]** | 2026 | 19 | 21 (HC) | 3T | SNpc/DGM | NM-only: NM volume / multimodal: N1 + NM + QSM | NR | NR | 0.86 / 0.99 |
| **Li Y et al. [22]** | 2026 | 26 (PD-RBD) | 46 (PD-noRBD; +48 HC) | 3T | SN | NM-related contrast | NR | NR | 0.69 |
| **Fang Y et al. [23]** | 2026 | 117 (TD-PD) | 43 r-ET / 94 nr-ET | NR | SN / LC | Multimodal: NM CNR + T1w/T2w ratio | NR | NR | 0.798 / 0.851 |
| **Chen T et al. [24]** | 2025 | 73 | 32 (PSP) | 3T | SNpc | SNpc volume (PD vs PSP) | NR | NR | 0.883 |
| **Shaff N et al. [25]** | 2024 | 31 | 22 (HC) | 3T | SN | Automated signal intensity | 86 | 83 | 0.81 |
| **Welton T et al. [26]** | 2024 | 82 | 107 (HC) | 3T | SN | Multimodal/AI: QSM-NMS composite; DL N1 models | 94.0; 100.0/83.95 | 86.0; 83.18/85.05 | 0.94; 0.916/0.898 |
| **Chen H et al. [27]** | 2024 | 73 | 65 (HC) | NR | SNpc | AI: QSM-derived NM-sensitive setMag (hybrid model) | 92.3 | 100.0 | Accuracy 96.3% / 95.8% |
| **Liu P et al. [15]** | 2024 | 30 | 50 HC | 3T | SNpc | NM-only: volume (PENCIL/MTC); CNR (PENCIL/MTC) | NR | NR | 0.943 / 0.934; 0.761 / 0.526 |
| **Lee DH et al. [28]** | 2024 | NR (195 total) | PET reference | NR | SN | Visual NM loss | NR | NR | 0.832 |
| **Seong M et al. [29]** | 2023 | 50 | 50 (HC) | 3T | Left SNpc (N1+N2) | Template-based CR | 98 | 94 | 0.994 |
| **Xuan T et al. [30]** | 2023 | 23 | 20 (ET) | NR | Middle SN | CNR | 65 | 87 | 0.7500 |
| **Hartono S et al. [31]** | 2023 | 80 | 111 HC | NR | SN | NM-only: contrast/size; multimodal: NM-iron composite | NR | NR | 0.86 / 0.83 / 0.94 |
| **Cao Q et al. [32]** | 2023 | 40 | 40 HC | NR | SN | Multimodal: SWI + NM volume + T2* R2* | 97.0 | 83.7 | 0.958 |
| **Oshima S et al. [33]** | 2023 | 22 | 22 non-PD (+22 HV) | NR | SN / LC | CR (NEX1 + dDLR) | NR | NR | 0.87 / 0.75 |
| **Liu Q et al. [34]** | 2023 | 10 early / 11 progressive PD | 10 HC | NR | LC | CNR | NR | 90.1 | NR |
| **Wang X et al. [35]** | 2023 | 43 TD-PD | 31 ET (+30 HC) | 3T | SN / LC | NM-only: combined SN/LC NM measures | NR | NR | 0.92 |
| **Gaurav R et al. [36]** | 2022 | 144 early PD | 55 HC | 3T | SNpc | NigraNet: Vol / Cvol / NSI / CNR | NR | NR | 0.83 / 0.85 / 0.79 / 0.77 |
| **Xing Y et al. [8]** | 2022 | 148 early PD | 97 HC | 3T | SNpc | NM-only: ventral NM contrast / volume | NR | NR | Accuracy 85% / 83% |
| **Oshima S et al. [37]** | 2021 | 25 | 49 (HC) | 3T | SNpc | Hyperintense area (DANTE, native space) | NR | NR | 0.94 |
| **Matsuura K et al. [38]** | 2021 | 244 PD | 49 PSP | 3T | LC | LC contrast ratio | NR | NR | 0.85 |
| **He N et al. [39]** | 2020 | 39 | 33 (HC) | 3T | SNpc | Multimodal: NM/R2* overlap percentage | NR | NR | 0.93 |
| **Liu XL et al. [40]** | 2020 | 18 | 15 (HC) | 3T | SN | NM-only/setMag comparison: SN hyperintense volume | 83.3/88.9 | 93.3/86.7 | 0.904/0.906 |
| **Shinde S et al. [41]** | 2019 | 45 | 35 (HC) | 3T | SNc | AI (NM-only): CNN-DL, held-out test set | 86 | 70 | 0.913 |
| **Jin L et al. [42]** | 2019 | 68 | 25 (ET) | 3T | SN | NM visual rating | 88.2 | 80.0 | 0.890 |
| **Pyatigorskaya N et al. [43]** | 2018 | 36 | 20 (HC) | 3T | SN | Multimodal: NM signal + volume + FA | 92 | 95 | 0.96 |
| **Taniguchi D et al. [44]** | 2018 | 24 PD | 10 HC (+11 PSP) | NR | SNpc | NM-SNpc area | 83 | 100 | 0.92 |
| **Xiang Y et al. [45]** | 2017 | 14 (PIGD) | 9 (TD) | 3T | Medial SNc | CR (ipsilateral to affected side) | 71.4 | 77.8 | 0.81 |
| **Schwarz ST et al. [46]** | 2017 | 39 | 30 (HC) | 3T | SNpc | Normalized NM volume (posterior/whole) | NR | NR | 0.92/0.88 |
| **Ehrminger M et al. [12]** | 2016 | 21 (iRBD) | 21 (HC) | 3T | LC/subcoeruleus | Visual signal intensity | 82.5 | 81.0 | NR |
| **Reimão S et al. [47]** | 2015 | 12 | 15 (ET) | 3T | SN | Area and width | 66.7 | 93.3 | NR |
| **Reimão S et al. [48]** | 2015 | 12 de novo / 10 early PD | 10 HC | 3T | lateral SN | Width | 88.5 / 75 | 65 / 75 | 0.806 / 0.77 |
| **Ohtsuka C et al. [49]** | 2014 | 30 | 10 (MSA-P) | 3T | Lateral SNc | CR (PD vs MSA-P) | 60 | 90 | NR |
| **Ogisu K et al. [50]** | 2013 | 18 | 27 (HC) | 3T | SNc | Semi-automated volume | 83 | 85 | 0.88 |
| **Ohtsuka C et al. [51]** | 2013 | 30 (early PD) | 22 (HC) | 3T | LC | CR (early PD vs HC) | 82 | 90 | NR |
| **Meta-analyses** | |  |  |  |  |  |  |  |  |
| **Cho SJ et al. [17]** | 2021 | 403 | 298 (HC) | NR | SNc | Pooled NM-MRI estimates | 89 | 83 | NR |
| **Wang X et al. [18]** | 2019 | NR | NR (HC) | NR | SNpc | Pooled NM-MRI estimates | 82 | 82 | NR |

Note. Only studies reporting at least one extractable numerical diagnostic or classification result are included. Values separated by slashes or semicolons correspond to the metrics, acquisition methods, models, or comparisons listed in the same order within that row. Sensitivity and specificity are percentages. Accuracy values are explicitly labelled in the final column and are not treated as AUC. Multimodal/composite results include NM-MRI as a prespecified model feature and should not be interpreted as NM-MRI-only performance. AI, artificial intelligence; AUC, area under the receiver operating characteristic curve; CNN, convolutional neural network; CNR, contrast-to-noise ratio; CR, contrast ratio; Cvol, intracranial-volume-corrected volume; DANTE, delay alternating with nutation for tailored excitation; dDLR, deep learning-based reconstruction; DGM, deep gray matter; DL, deep learning; ET, essential tremor; FA, fractional anisotropy; HC, healthy control(s); HV, healthy volunteer(s); iRBD, isolated rapid eye movement sleep behavior disorder; LC, locus coeruleus; MSA-P, multiple system atrophy with predominant parkinsonism; MT, magnetization transfer; MTC, magnetization transfer contrast; N1, nigrosome 1; NEX, number of excitations; NM, neuromelanin; NM-MRI, neuromelanin-sensitive magnetic resonance imaging; NMS, neuromelanin-sensitive; NR, not reported; NSI, normalized signal intensity; PD, Parkinson's disease; PD-RBD/PD-noRBD, PD with/without REM sleep behavior disorder; PENCIL, Proton-density Enhanced Neuromelanin Contrast in Low flip-angle gradient echo; PET, positron emission tomography; PIGD, postural instability/gait difficulty; PSP, progressive supranuclear palsy; QSM, quantitative susceptibility mapping; R2*, effective transverse relaxation rate; r-ET/nr-ET, essential tremor with/without resting tremor; ROI, region of interest; setMag, short-echo-time magnitude image derived from a QSM acquisition; SN, substantia nigra; SNc/SNpc, substantia nigra pars compacta; SPIR, spectral presaturation with inversion recovery; SWI, susceptibility-weighted imaging; T1w/T2w, T1-weighted/T2-weighted signal ratio; TD, tremor-dominant; TD-PD, tremor-dominant Parkinson's disease; Vol, volume.
